# Systemic nicotine exposure following switching to heated tobacco products: a category-level analysis of urinary nicotine equivalents

**DOI:** 10.1007/s11739-026-04562-0

**Published:** 2026-10-16

**Authors:** Jacqueline Miller‑Holt, Grant O’Connell, Maurane Charriere, Sylvain Larroque, Karin Jacobson

**Affiliations:** JT International SA, 8 Rue Kazem Radjavi, 1202 Geneva, Switzerland

**Keywords:** Meta-analysis, Nicotine, Heated tobacco, Bridging, Biomarkers of exposure, Tobacco harm reduction

Article 1(d) of the WHO Framework Convention on Tobacco Control recognizes harm reduction as a component of tobacco control. In practice, this involves the substitution of higher risk combustible tobacco products with non-combustible alternatives that substantially reduce exposure to harmful substances generated during combustion. Heated tobacco products (HTPs) are one such category of products. By heating rather than burning tobacco, HTPs avoid combustion, which is the primary source of the harmful substances present in cigarette smoke.

We previously introduced a structured and scientifically grounded framework for data “bridging” across the HTP category, which was published in this journal [1]. Our analysis demonstrated that adherence to the principle of heating, rather than burning, tobacco consistently results in substantial and comparable reductions in toxic emissions relative to combustible cigarette (CC) smoke, irrespective of product design, tobacco blend, additives, or heating technology. These reductions are mirrored by reductions in non-nicotine biomarkers of exposure, supporting the view that reduced toxicant exposure represents a category-level characteristic of HTPs rather than a product specific phenomenon. Independent assessments have subsequently reported similar conclusions, reinforcing the reproducibility and consistency of this category-level effect across diverse products, technologies and study designs [2].

Building on this framework, this commentary presents a secondary analysis of the same clinical evidence base identified by Miller Holt et al. (2026), extending the assessment from toxicant exposure and non‑nicotine biomarkers to nicotine exposure. Using urinary nicotine equivalents (NEq) as a biomarker proxy for systemic nicotine exposure, we provide a category-level view of nicotine exposure following HTP use across clinically evaluated studies (Fig. 1). Extending the toxicant exposure findings in Fig. 5 of Miller Holt et al. (2026)[1], we examined whether substantial reductions in toxicant exposure are associated with meaningful changes in nicotine exposure during actual product use. Figure 1 presents a descriptive analysis of NEq estimates by category (HTP and cigarette smoking) for each clinically evaluated product for which baseline and end-of-study mean values, standard deviation and sample size data were reported or could be derived (e.g. from SE, 95%CI). In addition, meta-analysis of absolute changes from baseline was performed to evaluate global estimates when combining results from multiple sources. The DerSimonian-Laird random-effects method was used with within-subject correlations defined, separately for CC and HTP, using available internal data and imputed to all the studies. Pooled estimates for HTP use demonstrated mean changes from baseline smoking of − 0.35 mg/g creatinine (95%CI [− 1.36;0.64]) and − 4.14 mg/24 h (95%CI [− 6.30;−1.98]) whereas the corresponding pooled estimates for continued cigarette use were +0.62 mg/g creatinine (95%CI [0.16;1.09]) and − 0.96 mg/24 h (95%CI [− 2.94;1.03]).

**Fig. 1:**
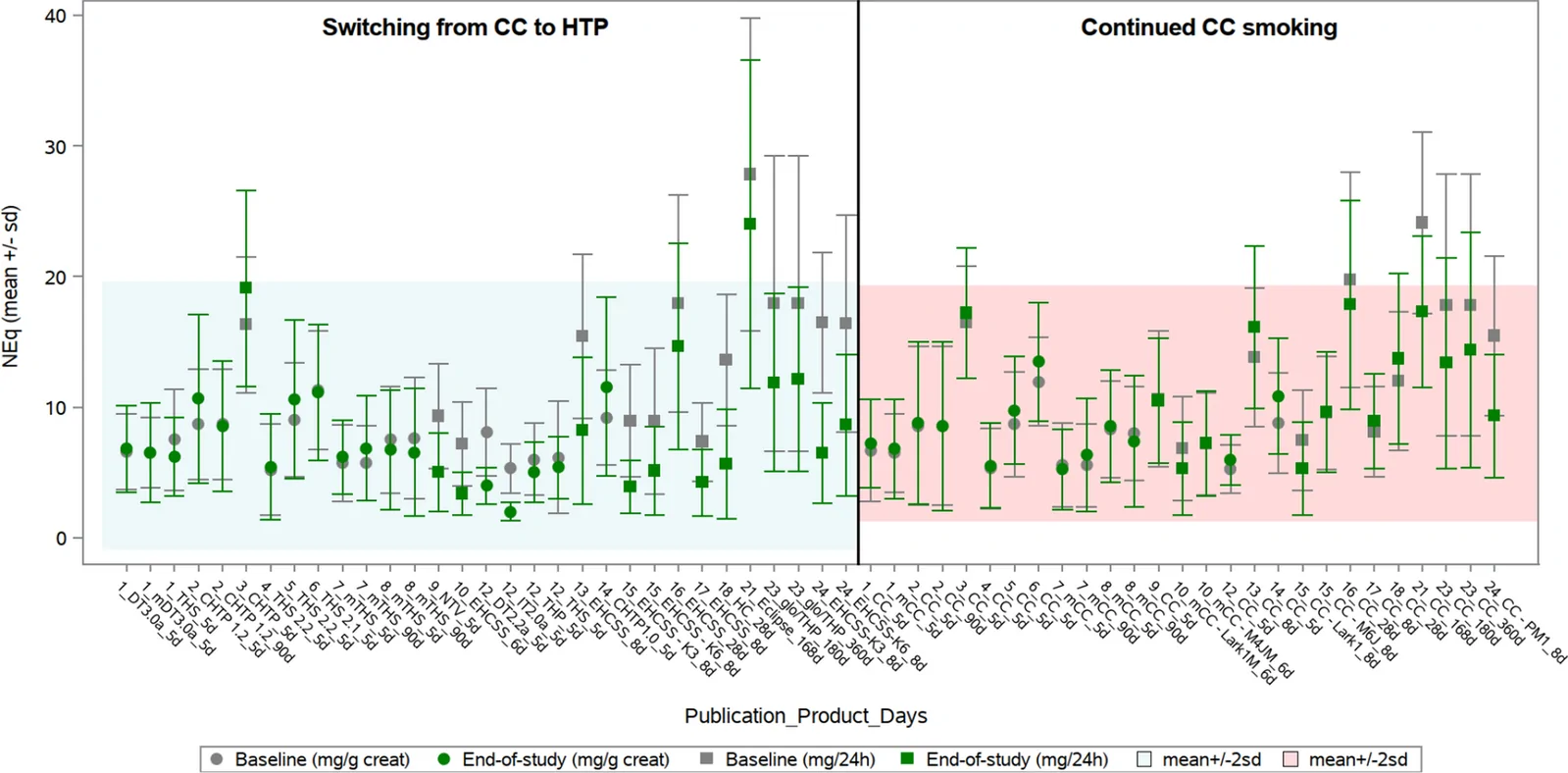
Nicotine equivalents (NEq) at baseline and end-of-study across clinically evaluated HTP switching and continued cigarette smoking studies included in the category-level assessment. Mean NEq (± SD). In all studies, NEq comprised nicotine and five major metabolites, except publication 21 in figure, which included nicotine and nine metabolites. Grey symbols represent baseline smoking and green symbols end-of-study (HTP use or continued smoking). Circles indicate creatinine-adjusted (mg/g creat) and squares non-creatinine-adjusted (mg/24 h). Shaded regions represent the category mean ±2 SD, calculated from all data points within each category and illustrating the overall distribution of nicotine exposure. *HTP* heated tobacco product; *CC* combustible cigarette

Despite differences in product design, heating technology, study populations and product use behaviors, a remarkably consistent category-level trend can be observed: when adults who smoke transition from cigarettes to HTPs, systemic nicotine exposure generally remains comparable to, or lower than, baseline cigarette smoking values. Across studies, mean end-of-study NEq values were also lower when switching to HTP (7.06 ± 2.64 mg/g creat and 9.45 ± 6.23 mg/24 h) than with continued smoking (8.03 ± 2.34 mg/g creat and 11.86 ± 4.42 mg/24 h). This consistency aligns with evidence indicating that most users adapt their behavior to obtain their preferred nicotine levels [3]. Although HTPs differ in heating technologies and nicotine delivery profiles, nicotine exposure remains broadly comparable to levels observed during cigarette smoking, irrespective of the specific HTP used.

While the underlying clinical studies exhibited methodological heterogeneity, the analysis was nevertheless able to identify a clear and consistent direction of effect across the evidence base. Across the evidence base, reductions in toxic emissions and non-nicotine biomarkers are accompanied by nicotine exposures that are generally lower than, or comparable to, those observed during cigarette smoking. Independent reviews and meta-analyses have reported similar findings when examining nicotine pharmacokinetics, concluding that non-combustible nicotine products are typically associated with lower overall nicotine exposures [4].

Occasional observations of marginally higher nicotine exposures should be interpreted in the broader context of harm reduction. For adults who smoke to transition away from combustible cigarettes, non-combustible alternatives must provide sufficient behavioral, sensorial and pharmacological satisfaction. Consequently, nicotine delivery that meets user preferences should not be considered inconsistent with harm reduction outcomes when accompanied by substantial reductions in toxicant exposure. The evidence considered here indicates that modest variation in nicotine exposure occurs within the context of a markedly reduced toxicant exposure profile [1]. Moreover, emerging evidence further suggests that substantial reductions in cigarette consumption is associated with lower toxicant exposures compared with people who exclusively smoke cigarettes [5].

Taken together, these findings extend the scientific basis for category-level assessment of HTPs. Our previous bridging analyses demonstrated substantial reductions in toxic emissions and biomarkers of exposure across clinically evaluated HTPs. The present analysis indicates that these reductions are not achieved at the expense of increased nicotine exposure, with systemic nicotine exposure generally remaining comparable to, or lower than, that observed during cigarette smoking. While long-term real-world evidence remains essential to understanding population-level impact, integrating robust bridging methodologies, with systematic post-market evidence generation and the emergence of category-level epidemiology will provide a scientifically coherent framework for evaluating HTP harm reduction impact. As demonstrated previously for snus in Sweden, it is ultimately the accumulation of category epidemiological evidence that will determine the extent to which reduced HTP exposure translates into measurable public health benefits.

## Supplementary Information

Below is the link to the electronic supplementary material.

Supplementary file1 (XLSX 261 KB)

## Data Availability

The datasets analysed in this study are provided within the supplementary information. Additional information can be obtained from the corresponding author upon request.
